# Genetic correction of induced pluripotent stem cells from a DFNA36 patient results in morphologic and functional recovery of derived hair cell-like cells

**DOI:** 10.1186/s13287-023-03617-9

**Published:** 2024-01-02

**Authors:** Yi Luo, Kaiwen Wu, Xiaolong Zhang, Hongyang Wang, Qiuju Wang

**Affiliations:** 1 Department of Audiology and Vestibular Medicine, Senior Department of Otolaryngology, Head and Neck Surgery, Chinese PLA Institute of Otolaryngology, the Sixth Medical Center of PLA General Hospital, 6 Fucheng Road, Beijing, 100048 China; 2 National Clinical Research Center for Otolaryngologic Diseases, Chinese PLA General Hospital, 28 Fuxing Road, Beijing, 100853 China; 3 Chinese PLA Medical School, 28 Fuxing Road, Beijing, 100853 China

**Keywords:** Induced pluripotent stem cells, *TMC1*, Hair cells, Differentiation, Whole-cell patch-clamp

## Abstract

**Background:**

*TMC1* is one of the most common deafness genes causing DFNA36. Patient-derived human induced pluripotent stem cells (iPSCs) provide an opportunity to modelling diseases. *TMC1* p.M418K mutation in human is orthologous to Beethoven mice. Here, we investigated the differentiation, morphology and electrophysiological properties of hair cell-like cells (HC-like cells) derived from DFNA36 patient.

**Methods:**

Inner ear HC-like cells were induced from iPSCs derived from DFNA36 (*TMC1* p.M418K) patient (M^+/−^), normal control (M^+/+^) and genetic corrected iPSCs (M^+/C^). Immunofluorescence, scanning electron microscopy and whole-cell patch-clamp were used to study the mechanism and influence of *TMC1* p.M418K mutation.

**Results:**

In this study we successfully generated HC-like cells from iPSCs with three different genotypes. HC-like cells from M^+/−^ showed defected morphology of microvilli and physiological properties compared to M^+/+^. HC-like cells from M^+/C^ showed recovery in morphology of microvilli and physiological properties.

**Conclusions:**

Our results indicate that *TMC1* p.M418K mutation didn’t influence inner ear hair cell differentiation but the morphology of microvilli and electrophysiological properties and gene correction induced recovery. CRISPR/Cas9 gene therapy is feasible in human patient with *TMC1* p.M418K mutation.

**Supplementary Information:**

The online version contains supplementary material available at 10.1186/s13287-023-03617-9.

## Introduction

According to the World Report on Hearing 2021 of the WHO, more than 1.5 billion people (nearly 20% of the global population) live with hearing loss; 430 million of them have disabling hearing loss. It is expected that by 2050, there could be over 700 million people with disabling hearing loss. Nearly 50–60% of deaf cases are caused by genetic etiology [1]. Sense of hearing relies on the conversion of mechanical stimuli induced by sound into neural signals which is conducted by inner ear hair cells. This process depends on the deflection of stereocilia bundles of hair cells opening the mechanoelectrical transduction (MET) channels [2]. Stereocilia are arranged in three rows with an increasing stepped pattern interconnected by several protein filaments, including the tip link that transfers mechanical stimuli to the MET channels. Several protein molecules have been identified as components of MET in hair cells, including cadherin 23 (CDH23), protocadherin 15 (PCDH15), transmembrane inner ear (TMIE), calcium and integrin-binding family member 2 (CIB2), lipoma HMGIC fusion partner-like 5 (LHFPL5) and transmembrane channel-like 1/2 (TMC1/2) [3]. In the past decade, TMC1/2 has emerged as pore-forming subunit of the MET [4, 5]. *TMC1* gene encodes TMC1 protein which is located on the surface of stereocilia. Mutations of *TMC1* result in both dominant (DFNA36) and recessive (DFNB7/11) non-syndromic hearing loss (NSHL) [6]. Following *GJB2, SLC26A4, MYO15A, OTOF* and *CDH23, TMC1* is the sixth most common genes causing deafness [7].

In 2002, DFNA36 was identified in a large North American family characterized with dominant, non-syndromic, bilateral, symmetric, sensorineural hearing loss that begins at 5–10 years old and rapidly progresses to profound deafness within 10–15 years [6]. The locus was mapped to human chromosome 9q11-q21 in a region overlapping the DFNB7/11 locus and this new dominant and recessive deafness-caused gene was named *TMC1* [6]. In the same year, Beethoven mouse with *Tmc1* p.M412K mutation was identified by ENU mutagenesis program, which showed progressive hearing loss and hair-cell degeneration similar to DFNA36 [8]. Our group found a large DFNA36 Chinses family with 222 members showing post-lingual, progressive and symmetric sensorineural hearing loss with *TMC1* p.M418K mutation [9] which is orthologous to *Tmc1* p.M412K mutation found in the Beethoven mouse model decade earlier [8]. The onset age ranged from 5 to 28 years old and high frequencies of hearing were initially affected with mild or moderated levels at the onset age and progressed to profound by the fifth or sixth decade [9]. In 2018, our group reported the second family with variant of *TMC1* p.M418K and the onset age of hearing loss were 8 and 20 years old [10].

Characteristics of late onset age and progressive hearing loss of DFNA36 make it a star model for gene therapy and several studies have achieve promising results. Gao and colleagues [11] injected Cas9-guide RNA-lipid complexes targeting the *Tmc1*^Bth^ allele into the cochlea of neonatal Beethoven mice. Remarkable alleviation of hearing loss and higher hair cell survival rates were observed in treated Beethoven mice. Gyorgy [12] screened 14 Cas9/gRNA combinations for specific and efficient disruption of Tmc1 mutant allele. A PAM variant of SaCas9-KKH that selectively and efficiently disrupted the mutant allele but not wildtype *Tmc1/TMC1* allele was identified in Beethoven mice and in a DFNA36 human cell line. Beethoven mice injected with adeno-associated virus (AAV2/Anc80)-mediated SaCas9-KKH delivery displayed lower hearing threshold, higher hair cell survival rate and preservation of normal hair bundle morphology [12]. Using dual delivery of SpCas9 and gRNA in separate AAV9-PHP.B vectors, Jason Wu [13] selectively disrupt *Tmc1* allele and preserves hearing in Beethoven mice up to 24 weeks. Besides the gene therapy targeting the mutant allele, selectively disrupting RNA of Tmc1 was another strategy. Using CRISPR/RfxCas13d editing system, which can specifically and precisely cleave single-strand RNAs, Zheng and colleagues reduced the *Tmc*^Bth^ transcript by 90.8% and improved hearing performance in both ABR and DPOAE threshold [14]. However, whether gene therapy for *TMC1* mutation-caused deafness is available for human beings remains unknown. Patients-derived induced pluripotent stem cells (iPSCs) offer a promising opportunity to understanding the pathogenic mechanisms and therapy methods in human. Gene therapy for iPSCs from patients with MYO7A, MYO15A and TRMU mutation have successfully rescued morphology and electrophysiological function of differentiated hair cells [15–17].

Previously, we have generated iPSCs from a patient carrying *TMC1* p.M418K mutation (M^+/−^) [18] and a gene-corrected iPSC line (M^+/C^) using CRISPR/Cas9 [19], as well as a normal control iPSCs (M^+/+^) from the patient’s normal son, who has no *TMC1* mutation and hearing loss. Here, we generated Hair Cell-like (HC-like) cells from iPSCs derived from genotypes of M^+/+^, M^+/−^ and M^+/C^ to investigate whether the *TMC1* p.M418K mutation may influence hair cell differentiation and gene correction could rescue the morphology and electrophysiological properties.

## Methods

### iPSCs culture

The iPSCs were generated from the family which has been reported by our team [9]. Urinary cells of the hearing loss patient with heterozygotic *TMC1* p.M418K (M^+/−^) and asymptomatic son with normal M^+/+^ were collected and induced to iPSCs [18]. *TMC1* mutation (p.M418K) was corrected with CRISPR/Cas9-mediated gene correction [19] (M^+/C^). iPSCs were cultured in Nuwacell hiPSC/hESCs medium (no. RP01020, Nuwacell) on matrigel (no. 354277, Corning) coated plates. At 80–90% confluency or 4–5 days, the cells were passaged with a split ratio of 1:5–1:20 using 0.5 mM EDTA (no. RP01007, Nuwacell).

### Hair cell differentiation

Differentiation protocol was referred to Ronaghi [20]. For embryoid body (EB) formation, the iPSCs were dissociated with 0.5 mM EDTA for 7–8 min at 37℃ and transferred to ultralow attachment surface six-well plates (no. 3471, Corning) in #1 medium: the hiPSC medium supplemented with 100 ng/mL recombinant human Dickopf-related protein (DKK-1; no. 5439, R&D Systems), specific inhibitor of smad3 (SIS3) at 3 μM (no. 566405, Sigma) and recombinant human insulin-like growth factors (IGF1; no. ab270062, Abcam) at 10 ng/mL (day 0). On day 15, EBs were transferred into poly-L-ornithine (no. P4957, Sigma) and matrigel (no. 354277, Corning)-coated plates and cultured for 3 days in #2 medium: the advanced DMEM/F12 supplemented with 20% knockout serum replacement (KSR; no. 10828028, Gibco), N2 (no. 17502048, Gibco), B27 (no. 17504044, Gibco), human bFGF at 25 ng/mL (no. 3718, R&D Systems), human FGF19 at 25 ng/mL (no. 969-FG, R&D Systems), human Noggin at 30 ng/mL (no. 6057, R&D Systems), human R-spondin 1 at 50 ng/mL (no. 4645-RS, R&D Systems), heperan sulfate at 50 ng/mL (no. 4777, Sigma) and ampicillin at 50 μg/mL (no. A5354, Sigma). On day 18, the medium was replaced with #3 medium: the advanced DMEM/F12 supplemented with 15% KSR, N2, and B27, human bFGF at 25 ng/mL, human FGF19 at 25 ng/mL, human BMP4 at 20 ng/mL (no. 314-BP, R&D Systems), heparan sulfate at 50 ng/mL, and ampicillin at 50 mg/mL. On day 21, the medium was replaced with #4 medium: the advanced DMEM/F12 supplemented with 15% KSR, N2, and B27, and ampicillin (50 mg/mL). The concentration of KSR was reduced to 10% on day 27 and to 5% on day 33. All the reagents were listed in Table 1.

**Table 1:** Reagents details

Reagent	Source	Identifier
*Chemicals, peptides, and recombinant proteins*		
hiPSC/hESCs medium	Nuwacell	Cat#RP01020
Matrigel	Corning	Cat#354277
0.5 mM EDTA	Nuwacell	Cat#RP01007
Recombinant human Dickopf-related protein	R&D Systems	Cat#5439
Specific inhibitor of smad3	Sigma	Cat#566405
Recombinant human insulin-like growth factors	Abcam	Cat#ab270062
Poly-L-ornithine	Sigma	Cat#P4957
Knockout serum replacement	Gibco	Cat#10828028
N2 supplement	Gibco	Cat#17502048
B27	Gibco	Cat#17504044
Human bFGF	R&D systems	Cat#3718
Human FGF19	R&D systems	Cat#969-FG
Human Noggin	R&D systems	Cat#6057
Human R-spondin 1	R&D systems	Cat#4645-RS
Heperan sulfate	Sigma	Cat#4777
Ampicillin	Sigma	Cat#A5354
Advanced DMEM/F12 medium	Gibco	Cat#12634010
Human BMP4	R&D systems	Cat#314-BP
FM1-43	Invitrogen	Cat#35355
*Antibodies*		
EYA1	Invitrogen	Cat#PA5-65034
TFAP2A	Abcam	Cat#ab52222
PAX2	Abcam	Cat#ab79389
PAX8	Proteintech	Cat#60145-4-Ig
MYO7A	Abcam	Cat#155984
ATOH1	Proteintech	Cat#21215-1-AP
POU4F3	Proteintech	Cat#21509-1-AP
Tubulin	Abcam	Cat#ab195884
F-actin	Abcam	Cat#ab130935
TMC1	Abcam	Cat#ab199949
Goat anti-Rabbit IgG, Alexa Fluor™ 555	Invitrogen	Cat#A-21428
Goat anti-Mouse IgG, Alexa Fluor™ 488	Invitrogen	Cat# A-11001

### Immunofluorescence

Cells were collected and transferred and attached to confocal dishes. Cells were fixed with 4% PFA for 30 min at room temperature. Samples were permeabilized and blocked in PBS containing 0.3% Triton X-100 (Sigma) and fetal bovine serum (Sigma) for 1 h at room temperature. Cells were incubated with primary antibodies for 1 h at room temperature. After washing with PBS for 3 times, specimens were incubated with secondary antibodies for 1 h at room temperature. Images were acquired by Carl Zeiss LSM980. Antibodies were listed in Table 1.

### FM1-43 staining

FM1-43 was reconstituted in ice-cold HBSS to working staining solution of 5 μg/mL. Culture medium was aspirated and cells were rinsed in staining solution on ice for 1 min. The sample was imaged in staining solution immediately using Zeiss LSM980 with the same condition.

### Scanning electron microscopy (SEM)

HC-like cells were fixed with 2.5% glutaraldehyde at 4 ℃ for 2 h. Cells were washed with PB for 3 times and then fixed with 1% osmium tetroxide for 30 min. After washed with PB 3 times, cells were incubated in 2% tannic acid for 30 min. Cells were washed 3 times again and then dehydrated with a gradient concentration of ethanol (50%, 70%, 90% and 100%). The specimens were then immersed in isoamyl acetate, dried with a critical point dryer, coated with Aurum (Hitachi E102) and then imaged at 3 kV with a scanning electron microscope (Hitachi SU8600 FESEM).

### Electrophysiology

The membrane currents of iPSC-derived HC-like cells were measured using the whole-cell patch-clamp technique with an amplifier (EPC10; HEKA). Data was recorded by Patchmaster software and analyzed by Igor software. For I_K_ and I_K1_, extracellular solution contained 137 mM NaCl, 4 mM KCl, 1.8 mM CaCl_2_, 1 mM MgCl_2_, 10 mM Glucose, 10 mM HEPES (pH 7.4), and the intracellular (pipette) solution contained 30 mM KCl, 110 mM K-Glucobate, 2 mM MgCl_2_, 1 mM EGTA, 10 mM HEPES, 4 mM Mg-ATP, 0.3 mM Na-GTP (pH 7.2). For I_Ca_, extracellular solution contained 140 mM TEA-Cl, 4 mM KCl, 1 mM MgCl_2_, 10 mM HEPES, 10 mM CaCl_2_, 5 mM Glucose (pH 7.4), and the intracellular (pipette) solution contained 110 mM CsCl, 1 mM CaCl_2_, 5 mM HEPES, 10 mM EGTA, 4 mM Na2-ATP, 4.5 mM Mg-ATP (pH 7.2). I_K1_ current was recorded in voltage-clamp mode with a start holding potential of − 70 mV and incremental steps of 10 from − 70 to − 140 mV, and I_K_ current was recorded with incremental steps of 10 mV up from − 70 to 20 mV, and I_Ca_ current was recorded with incremental steps of 10 mV from − 70 to 70 mV. AP current was recorded with current-clamp mode with a start holding current of 0 pA and incremental steps of 10 pA from − 50 pA to 240 pA.

### Real-time PCR

Total RNAs were obtained from cells using RNAeasy™ Animal RNA Isolation Kit with Spin Column (Beyotime, no. R0026) and reverse-transcribed into cDNA using iScript™ cDNA Synthesis Kit (Bio-Rad, no. 1708891). RT-qPCR was performed on the Bio-Rad CFX96. These data were analysed using BioRadCFXManager and relative gene expressions were calculated using the 2^−△△Ct^ method using ACTB as a housekeeping gene. Primers for RT-qPCR were listed in Additional file 1: Table S1.

### Statistical analysis

Statistical analysis was performed using Graphpad Prism (Version 8.0.2). The mean values were statistically compared using an two-way ANOVA for electrophysiological analysis. One-way ANOVA was used to compare. One-way ANOVA was performed for analysis of lengths, diameters and densities of microvilli. Turkey multiple comparison test was applied to compare the difference in groups. *P* values of less than 0.05 were considered to be statistically significant.

## Results

### Differentiation of iPSCs into otic progenitors and inner ear hair cell-like cells

**Fig. 1:**
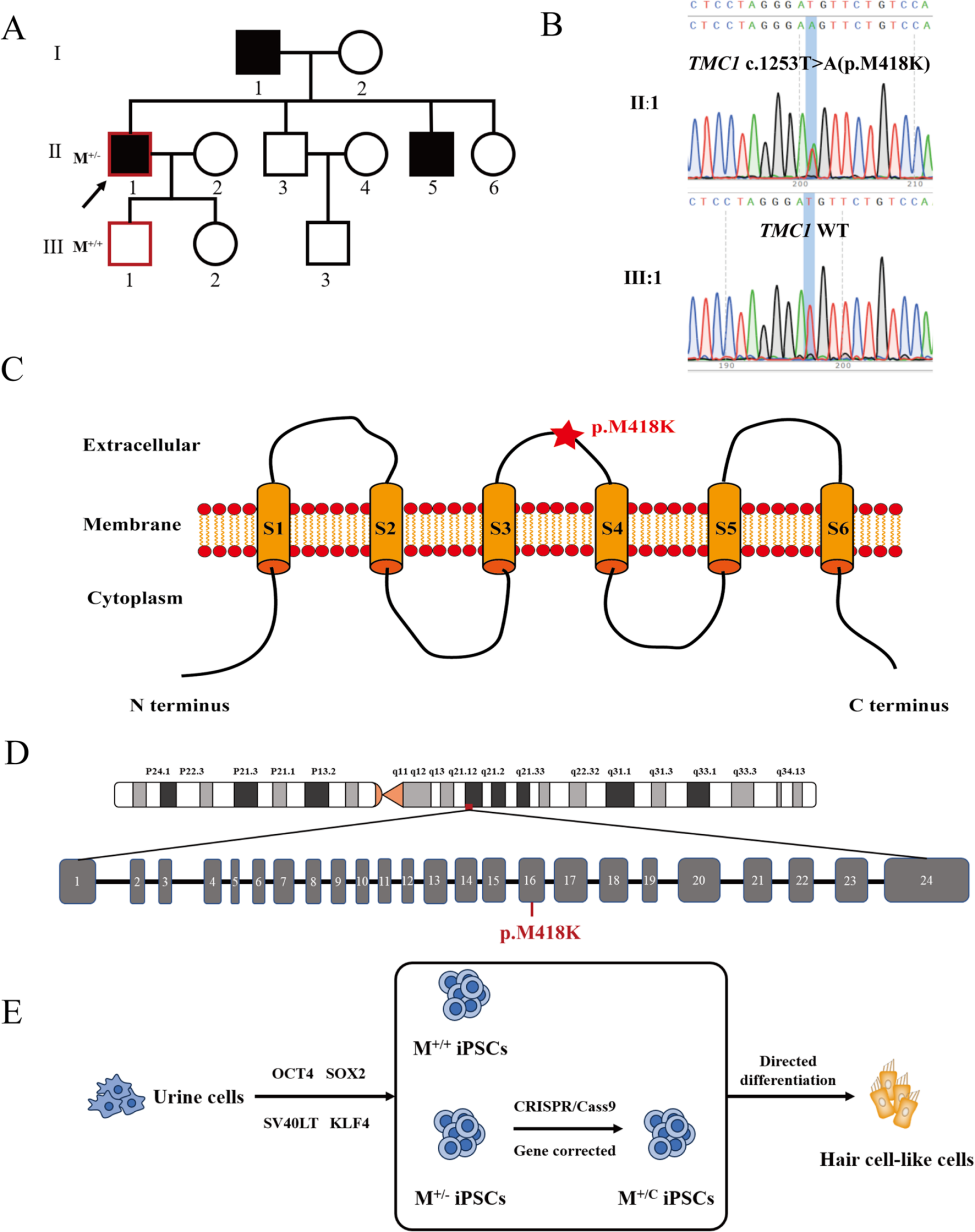
Generation of iPSCs from patient with *TMC1* p.M418K mutation. **A**: Portion of one Chinese pedigree family reported previously, patient (M^+/−^) and wild-type (M^+/+^) were indicated. Affected subjects are denoted in black. Proband is indicated by an arrow. Symbols with red frame indicate members whose cells were reprogrammed to iPSCs. **B**: Sanger sequencing of *TMC1* p.M418K mutation from II:1 and WT from III:1. **C**. Schematic diagram of TMC1 protein structure which contains 6 transmembrane helixes. The p.M418K mutation locates in the second extracellular domain. **D**: Schematic of *TMC1* locus on the 9q21 chromosomal region and structure of *TMC1* gene which has 24 exons. The M418K mutation is in exon 16. **E**: Schematic illustrating the generation and differentiation of iPSCs from patient, WT control and CRISPR/Cas9 corrected cell lines. M^+/−^, patients with *TMC1* p.M418K; M^+/+^, normal control from the Family with no *TMC1* p.M418K mutation; M^+/C^, iPSC from patients with *TMC1* p.M418K corrected by CRISPR/Cas9

**Fig. 2:**
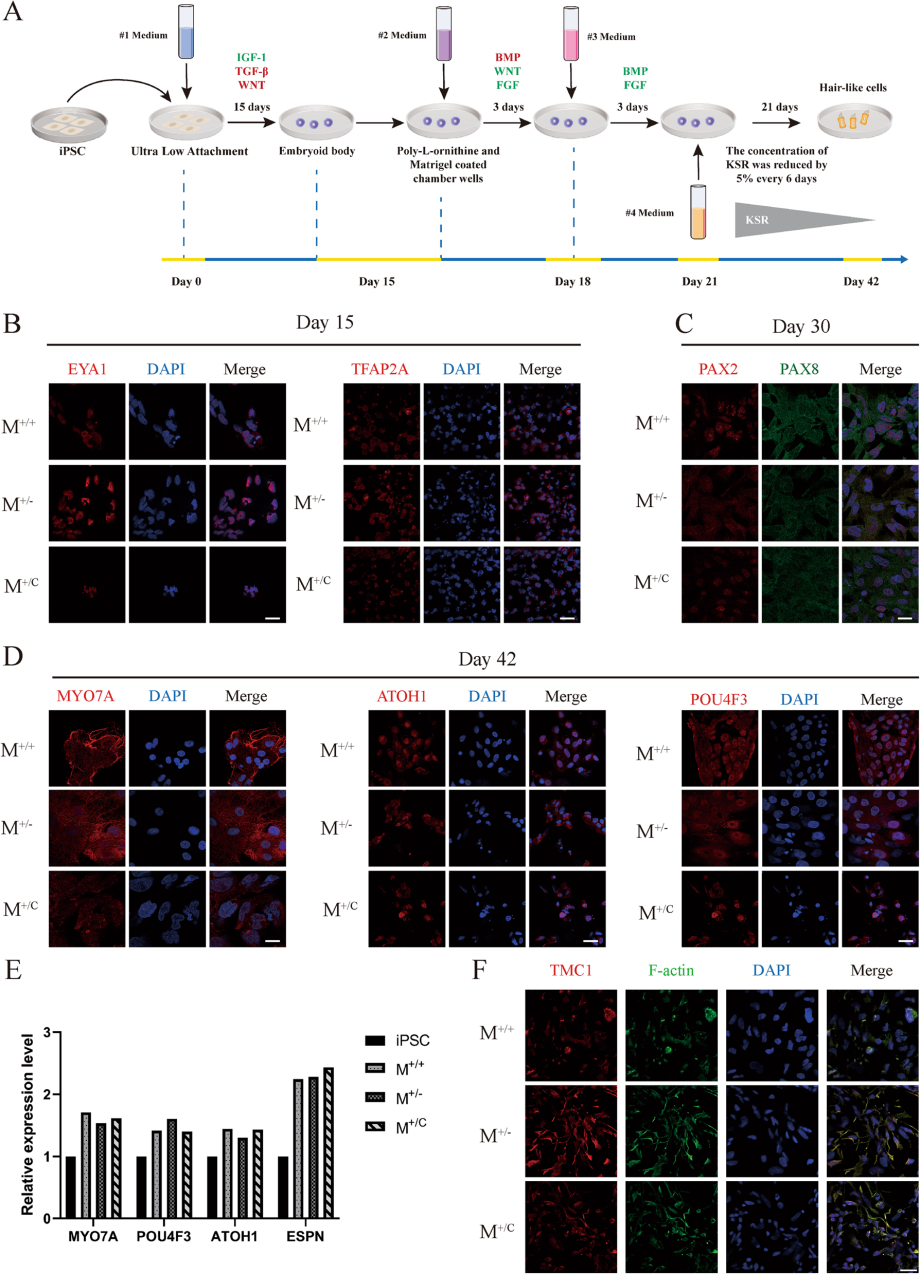
Differentiation into otic progenitor cells and hair-like cells. **A**: Schematic diagram illustrating differentiation protocol for iPSC-derived hair-like cells. **B**: iPSCs from different genotype were induced toward NNE on day 15. Cells were stained with NNE markers EYA1 and Tfap2α. Scale bar: 25 μm. **C**: Otic progenitors were induced from different genotype iPSCs on day 30 and were stained with specific markers Pax2 (red) and Pax8 (green). Scale bar: 25 μm. **D**: iPSC-derived HC-like cells from different genotype on day 42 were identified with markers MYO7A, ATOH1 and POU4F3. Scale bar: 25 μm. **E**: Relative mRNA expression levels of hair cell markers MYO7A, POU4F3, ATOH1 and ESPN were analyzed using SYBR green real-time PCR and were normalized to ATCB mRNA expression. **G**: TMC1 protein was expressed on membrane of HC-like cells and co-expressed with F-actin. Scale bar: 25 μm

We generated three iPSC lines from patient II:1 and his son III:1: *TMC1* p.M418K (M^+/−^), normal control (M^+/+^) and gene corrected iPSCs (M^+/C^) (Fig. 1A and E). The genotypes were confirmed by Sanger sequence (Fig. 1B). All three iPSCs were induced into non-neural ectoderm (NNE) on day 15 with activation of IGF1 and inhibition of TGF-β and WNT pathways. The differentiation of NNE was defined by immunostaining with antibodies specific for EYA1 and Tfap2α (Fig. 2B). On day 30, by inhibition of BMP and activation of WNT and FGF for 3 days, followed by activation of BMP and FGF for another 3 days, the otic progenitors were identified with specific antibodies for PAX2 and PAX8 in all three groups cells (Fig. 2C). On day 42, after culturing with N2, B27 and knockout serum replacement for 24 days, cells derived from M^+/+^, M^+/−^ and M^+/C^ all showed ATOH1, MYO7A and POU4F3 expression (Fig. 2D). The brightfield images of iPSCs, embryoids and HC-like cells were presented in Additional file 1: Fig. S1 and the morphology of HC-like cells is significantly different from their iPSC lines. Real-time PCR confirm the MYO7A, POU4F3 ATOH1 and ESPN expression compared to iPSCs (Fig. 2E). Meanwhile, TMC1 protein were detected in HC-like cells membrane and co-expressed with F-actin, which indicated that TMC1 protein is located in microvilli (Fig. 2F). We performed sanger sequencing on HC-like cells derived from three iPSC lines and they all carried the same genetic background with iPSCs (Additional file 1: Fig. S2). There was no difference in cell proliferation among the three HC-like cell lines (Additional file 1: Fig. S3). Therefore, these results demonstrated that all iPSCs derived from patient, normal control, and gene-corrected iPSCs could differentiate into otic progenitors and HC-like cells.

### Abnormal microvilli morphology of inner ear HC-like cells

**Fig. 3:**
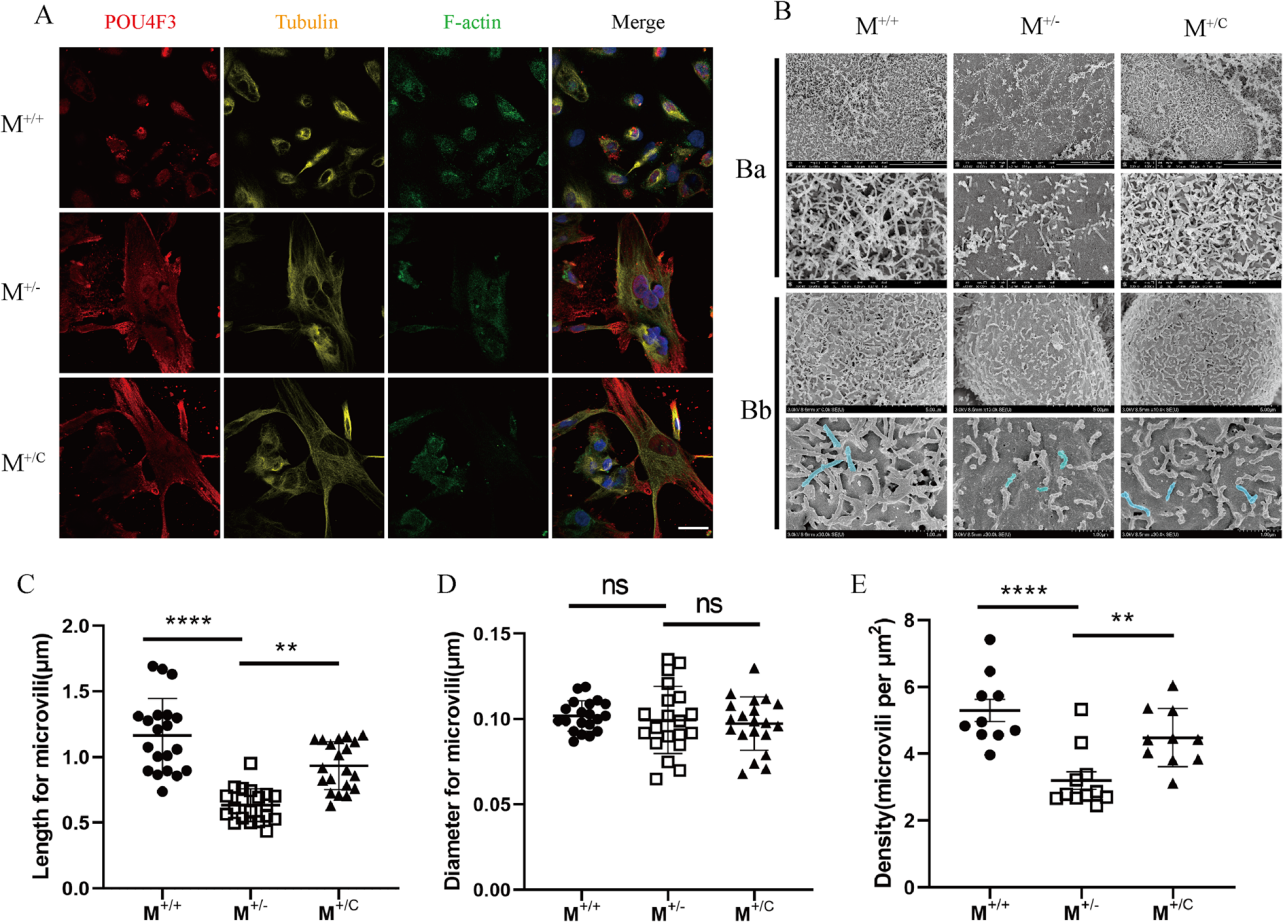
Abnormal morphology of inner ear HC-like cells. **A**: iPSC-derived hair-like cells on day 42 showed hair bundle-like protrusions observed by Tubulin and F-actin using laser scanning confocal microscopy. Scale bar: 25 μm. **B**: SEM of HC-like cells derived from iPSCs on day 45. Microvilli structure could be observed. Ba showed microvilli of multi-layer hair-like cells. Bb showed microvilli of single-layer hair-like cells and typical microvilli indicated by pseudocolor. **C**–**E**: The length (**C**) (*n* = 20), diameter (**D**) (*n* = 20) and density (**E**) (*n* = 10) were measured by ImageJ software. **P < 0.005; ****P < 0.0001. ns: no significance

In order to investigating the morphology of stereocilia-like protrusions in iPSC-derived HC-like cells, we proceeded SEM technique to analyze the cilialium. Immunofluorescence revealed that all three iPSCs-derived HC-like cells that expressed POU4F3 showed positive Tubulin and F-actin (Fig. 3A), which indicated that microvilli presented in all three cell lines. SEM revealed abnormal morphology of p.M418K-derived HC-like cell (Fig. 3B). Figure 3Ba showed microvilli of multi-layer and hair cells derived from M^+/−^ presented sparser and shorter. However, microvilli of hair cells derived from M^+/+^ and M^+/C^ showed denser and longer. Microvilli of single hair cell were imaged for further statistical analysis and the typical microvilli were indicated by pseudocolor (Fig. 3Bb). We used Image J to assess the effects of p.M418K mutation on the morphological properties of HC-like cells on the length, diameters and density of hair-like microvilli structure. The average lengths (n = 20) of microvilli in M^+/+^, M^+/−^ and M^+/C^ HC-like cells were 1.164, 0.634 and 0.934 μm (Fig. 3C), the average diameters (n = 20) in those cells were 0.102, 0.100 and 0.097 μm (Fig. 3D), the average densities (n = 10, microvilli per μm^2^) in those cells were 5.298, 3.200 and 4.487 (Fig. 3E), respectively. These results indicated that *TMC1* p.M418K mutation affected microvilli of inner ear HC-like cells in length and densities.

### Defects of p.M418K-derived HC-like cells in electrophysiology

**Fig. 4:**
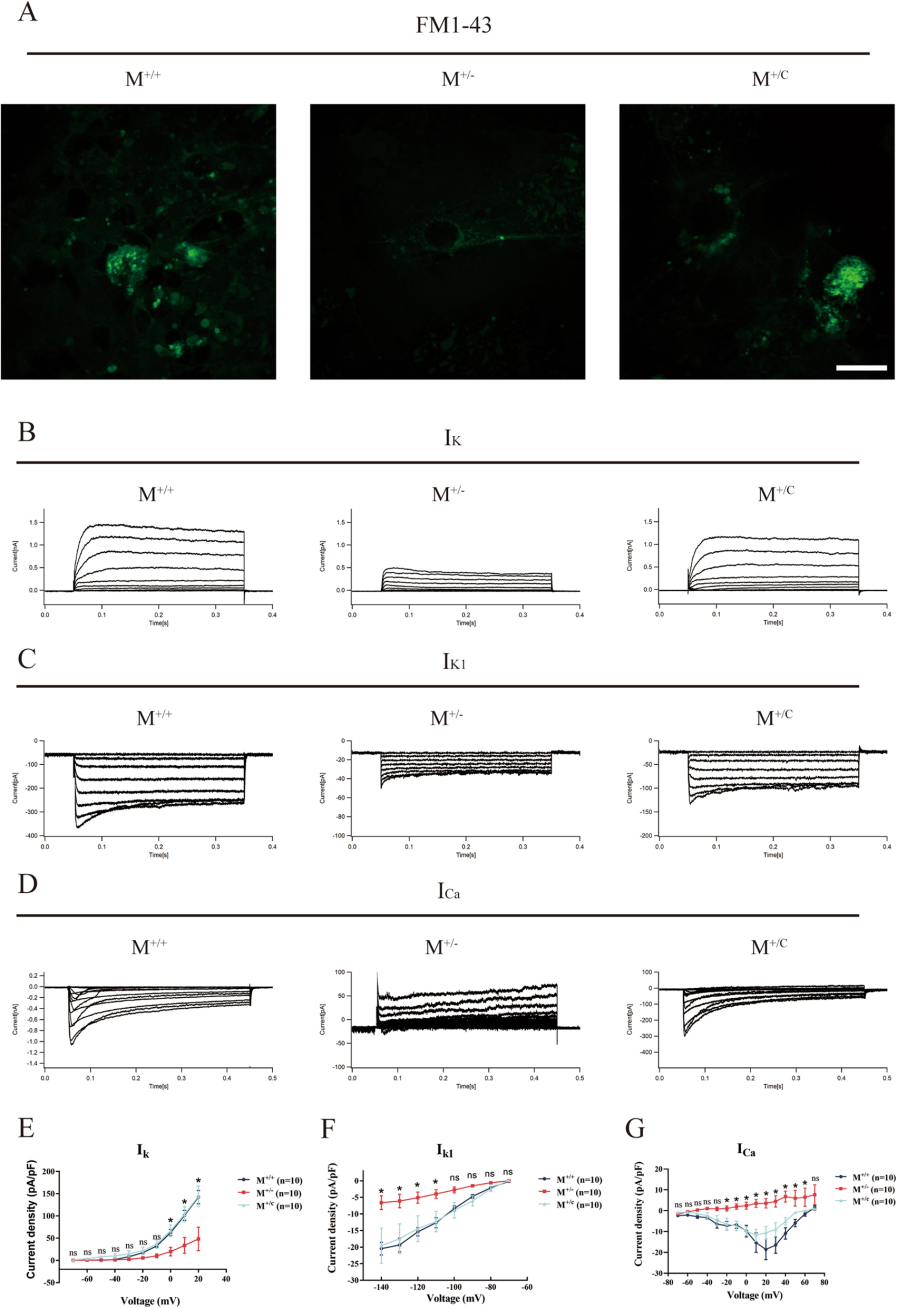
Electrophysiological activity of HC-like cells induced from iPSCs using whole-cell patch-clamp technique. **A**: FM1-43 dye in HC-like cells induced from iPSC of M^+/+^, M^+/−^ and M^+/C^ on differentiation day 42. Scale bar = 25 μm. **B**–**D**: Representative currents diagram of I_K_ (**B**), I_K1_ (**C**) and I_Ca_ (**D**) on day 43–45. **E**–**G**: Further statistical analysis based on the recordings. Current density was calculated as current intensity (pA) divided by C-slow (pF) (*n* = 10). Data are displayed with mean ± SEM. ns for no significant, *P < 0.05 by one-way ANOVA followed unpaired Student’s t test; ns: no significance

HC-like cells were then stained with FM1-43 to assess the function of ion channels. M^+/−^-derived HC-like cells lacked the ability of uptaking of FM1-43, compared to its M^+/+^ control. M^+/C^-derived HC-like cells showed partially recovery of the ion channel function of uptaking FM1-43 (Fig. 4A). To assess the electrophysiological properties of HC-like cells, voltage-dependent outward K^+^ (I_K_), inward K^+^ (I_K1_) and inward Ca^2+^ (I_Ca_) were measured using whole-cell patch-clamp technique, which represented specific electrophysiological properties of inner ear hair cells. Figure 4B–D show the representative I_K_, I_K1_ and I_Ca_ currents waveforms recorded from M^+/+^, M^+/−^ and M^+/C^-derived HC-like cells. Compared to M^+/+^, M^+/−^ showed significant lower currents in outward I_K_ currents, inward I_K1_ currents and completely vanish of I_Ca_ currents (Fig. 4B–D). While, those currents in gene-corrected M^+/C^-derived HC-like cells were comparable with those in the M^+/+^ HC-like cells (Fig. 4B–D). Further, currents density was calculated as current intensity (pA) divided by C-slow (pF). The current- voltage curves were assessed based on the recordings. In the I_K_ current densities (pA/pF) in three HC-like cells of different genotypes, significant variations displayed at depolarizing steps ranging from 0 to 20 mV (Fig. 4E). At hyperpolarizing steps from -100 to -140mv, the average I_K1_ current densities (pA/pF) also showed significant difference in HC-like cells from iPSCs of three genotypes (Fig. 4F). As for the I_Ca_ current densities (pA/pF), significant variation was observed at depolarizing and hyperpolarizing steps from − 20 to 50 mV (Fig. 4G). These results demonstrated that *TMC1* p.M418K mutant defects in electrophysiological properties of HC-like cells derived from iPSCs.

### Effects of gene correction on morphology and function recovery of HC-like cells

CRISPR/Cas9 was used to correct the *TMC1* p.M418K mutation in the iPSC line and the pluripotency was identified[19]. M^+/C^ iPSCs would differentiate into NNE (Fig. 2B), otic progenitors (Fig. 2C) and HC-like cells (Fig. 2D). The HC-like cell derived from M^+/C^ iPSCs have microvilli (Fig. 3A–B). The length and density of microvilli in HC-like cells partially recovered compared to that derived from M^+/−^ iPSCs (Fig. 3B–E). As for the electrophysiological properties, HC-like cells derived from M^+/C^ show partially recovery of FM1-43 uptake (Fig. 4A), I_K_, I_K1_ and I_Ca_ (Fig. 4B–G). Gene editing didn’t interfere the differentiation of HC-like cells and partially restored the electrophysiological properties.

## Discussion

Hereditary hearing loss is a common disabled disease which accounts for over 50%-60% of childhood hearing loss [1]. With the widespread newborn hearing screen combined with genetic testing, increasing genetic hearing loss has been identified in the early stages after birth [21–23]. Hearing aids (HAs) and cochlear implants (CIs) are main hearing rehabilitation methods for hearing loss patients. While HAs and CIs are effective, they do not actually restore ‘biologically normal’ hearing level. Thus, novel biological therapies like stem cell therapy and gene therapy have emerged as promising therapeutics to restore or prevent hearing loss [24]. CRISPR/Cas9 system has been a widely applied gene-edit tool because of its clear targeting, short RNA sequences and simultaneous operation of multiple genetic loci [25]. Even though gene therapy for various system like brain, spinal cord, liver, eyes and muscle have achieved notable progression in clinical trials [26], none clinical trials of gene therapy in inner ear is initiated.

In the field of gene therapy in inner ear, gene replacement [27, 28], gene suppression using antisense oligonucleotides [29, 30] or RNA interference [31, 32], and CRISPR/Cas9 based gene editing [11, 33–35] have achieved encouraging progress in animal models, but there is a long way before they are applied in clinic. Several non-gene-edit methods have been applied for *Tmc1*-associated hearing loss. RNAi-mediated gene silencing carried by AAV vectors can alleviate hearing loss, improve hair cell survival and prevent stereocilia bundle degeneration in dominant Beethoven mice [32, 36]. Therapeutic method combining RNAi and gene replacement also achieved robust hair cell and hearing level preservation in Beethoven mice [37]. As for the recessive *Tmc1* mutant mice, gene replacement was applied to partially recovers hearing level and balance outcomes [13, 27]. However, RNAi and gene replacement may require repeated injection to maintain a long-term effect [38]. The majority of Beethoven mice injected with single injection of RNAi maintained hearing acuity for 26 weeks [32]. Thus, gene edit that can correct gene-type permanently may resolve this obstacle. CRISPR/Cas9-based gene therapy had rescued hearing and hair cell survival in Beethoven mice [11–14] and recessive mice with *Tmc1* p.Y182C mutation [13, 33]. Our group found a large family with *TMC1* p.M418K mutation which is orthologous to *Tmc1* p.M412K mutation in Beethoven mouse [9] in 2014 and identified a novel likely pathogenic variant c.797 T > C and the second family of p.M418K in 2018 [10]. The Beethoven mice which is orthologous to *TMC1* p.M418K in human also shows progressive hearing loss, hair cell decreasing and hair bundle disorganization [6, 11, 12]. The progressive hearing loss of patients with *TMC1* p.M418K gives us a time window for gene therapy after birth. We have generated iPSCs from patient with *TMC1* p.M418K mutation, and then performed gene correction [18, 19].

In the present study, based on three iPSC lines (M^+/+^, M^+/−^ and M^+/C^) derived from a progressive deaf patient with *TMC1* p.M418K mutant and his normal son, we generated HC-like cells of three genotypes (Figs. 1E and 2A). All three iPSCs have been identified with pluripotency, which indicated that the *TMC1* p.M418K mutations did not influence the induction and pluripotency of iPSCs[18, 19]. Cells derived from all three iPSC lines presented the same characteristics when induced into NNE, otic progenitors and HC-like cells, which proof that TMC1 is not required for the differentiation of otic cell lines (Fig. 2). These results are consistent with previous researches on *TMC1* p.M418K mutation patients and Beethoven mice. *TMC1* p.M418K mutation patients have normal hearing level at birth but show post-lingual, bilateral, symmetric sensorineural hearing loss[9]. Patients-derived iPSCs offer a promising opportunity for researching diseases of pathogenic mechanism and treatment, such as hereditary deafness. In 2006, by introducing four factors, Oct3/4, Sox2, c-Myc and Klf4, Shinya Yamanaka produce iPSC from mouse embryonic or adult fibroblasts[39]. In the next year, iPSC was generated from adult human fibroblasts[40]. Because of its pluripotency, non-immunogenic and ethical issues-free associated with human embryonic stem cells, iPSCs have been widely applied in various diseases for pathogenic mechanism and therapeutics. iPSCs offer advantages for disease modelling and therapy methods. FDA Modernization Act 2.0’s emphasised on alternative non-animal testing methods, thus iPSCs-based modelling and drug development for diseases serve as an animal-free preclinical test model[41]. iPSC-based researches have achieved massive inspiring fruition in various diseases like amyotrophic lateral sclerosis[42], Huntington's disease[43], blood diseases[44] and spinal muscular atrophy[45]. Using patient iPSCs-derived cortical spheroids, the mechanism of PSEN1 L435F mutation affecting neurodevelopment by increasing Notch signalling in familial Alzheimer’s disease was revealed[46]. Injection of islets derived from human iPSCs effectively restored endogenous insulin secretion and improved glycemic control in non-human primates[47]. In the field of inner ear, methods of inner ear hair-like cell induction have been established by several protocols[48] and may become important platforms to tackle hereditary sensorineural hearing loss in combination with targeted genome editing[49]. Studies of iPSCs from *MYO15A* and *MYO7A* mutation patients demonstrate the feasibility of generating inner ear hair cells from human iPSCs and the functional rescue of gene mutation-caused hearing loss by using genetic correction[15, 17].

HC-like cells were identified with F-actin and Tubulin labelling microvilli from all three groups (Fig. 3A), indicating the *TMC1* p.M418K mutation and gene correction have no influence on the appearance of microvilli. Nevertheless, the morphology of microvilli of HC-like cells derived from M^+/−^ differed from those of the M^+/+^ and M^+/C^ (Fig. 3B-E). HC-like cells carried *TMC*1 p.M418K mutation presented normal cell proliferation compared with its WT control (Figure S3). This may be attributed to the difference between cells cultured in vitro and those in vivo. The abnormal morphology of microvilli may be attributed to the dominant *TMC1* p.M418K mutation. Beethoven mice and human patients with *TMC1* p.M418K mutation show a phenotype with progressive hearing loss[8, 9]. The loss of hair cells and severe disorganization of hair bundles were major pathogenic characteristics of Beethoven mice[8]. Our results proof the defects of hair bundle-like microvilli in human patient-derived HC-like cells with *TMC1* p.M418K mutation. Studies on other pathogenic genes also demonstrated defected microvilli or stereocilia-like protrusions derived from iPSC. The morphology of the stereocilia-like protrusions of HC-like cells derived from compound heterozygous MYO7A mutations (c.1184G > A and c.4118C > T) displayed significant differences from those of cells from normal control[15]. Compound heterozygous MYO15A (c.4642G > A and c.8374G > A) and double mutation of TRMU (c.28G > T) and mitochondrial DNA mutation (12S rRNA, m.1555A > G) also demonstrated defected morphology of stereocilia-like protrusions compared to normal control[16, 17]. However, all of them showed the recovery of the morphology of protrusions by gene correction.

As for the electrophysiological activity, M^+/−^-derived HC-like cells also presented deficiencies in uptaking FM1-43 dye and partial recovery was observed in M^+/C^-derived HC-like cells (Fig. 4A). We demonstrated the impact of *TMC1* p.M418K mutation on HC-like cells with voltage-dependent outward K^+^ currents (I_K_), inward K^+^ currents (I_K1_) and inward Ca^2+^ currents (I_Ca_). M^+/−^-derived HC-like cells exhibited significant defects in electrophysiological properties, including decreasing current densities of I_K,_ I_K1_ and completely depletion of I_Ca_ (Fig. 4E). Gene-corrected HC-like cells exhibited complete recovery of I_K,_ I_K1_ and I_Ca_. These results demonstrated that *TMC1* p.M418K mutation impaired the ion channels of HC-like cells of patient-iPSCs, especially in I_Ca_. This is consistent with previous studies which proof *TMC1* is a cation channels and has a prior permeability to calcium ions [4, 5, 50]. Hair cells of mouse with mutant *Tmc1* had reduced calcium permeability and reduced single-channel currents, which demonstrated TMC1 is components of hair cell transduction channels and contribute to permeation properties [50]. In HC-like cells derived from iPSCs with mitochondrial DNA mutation (12S rRNA, m.1555A > G) and TRMU mutation (c.28G > T), current densities of I_K_ and I_K1_ were significant impaired rather than that of I_Ca_ [16], indicating the differences in electrophysiological properties due to different genes. Furthermore, studies proofed that TMC1 is a pore-forming component of mechanosensitive ion channels in auditory hair cells [4, 5]. Our results provide evidences that *TMC1* plays the same role in transduction channel of hair cells in human with iPSC-derived HC-like cells and gene correction could be applicable in patients with *TMC1* mutations.

However, limitation exists in this study. Similar to previous researches applied by other groups [15–17, 51], gene editing was performed on iPSCs instead of differentiated mature HC-like cells. Gene therapy should be performed before lesion occurs, but defects of microvilli and electrophysiological function appeared initially in vitro instead of progressive damage in vivo. For this reason, it’s hard to choose the right time to perform gene therapy on HC-like cells. Inner ear organoid derived from iPSCs which is more similar to primary inner ear may conquer this obstacle [51–53].

## Conclusions

*TMC1* p.M418K mutation influence the microvilli and ion transduction channels in inner ear HC-like cells induced from iPSCs. Gene correction by CRISPR/Cas9 induced the recovery of the morphology and electrophysiology properties of HC-like cells derived from *TMC1* p.M418K iPSCs, which proofed that gene correction was a promising therapy in human. HC-like cells derived from iPSC with *TMC1* p.M418K provide a model of hereditary progressive hearing loss in cellular level, which would play an essential role in pathogenesis and therapeutics in an animal-free way.

### Supplementary Information

**Additional file 1. Table S1**. Primers. **Figure S1**. The brightfield images of iPSCs, embryoid and HC-like cells. The morphology of HC-like cells is significantly different from their iPSC lines. Scale bar: 200 μm. **Figure S2**. Sanger sequencing of HC-like cells. Sanger sequencing of HC-like cells derived from three cell lines showed same gene background with iPSCs. **Figure S3**. Cell proliferation curve. The A450 on 0, 24, 48 and 72 hours of HC-like cells derived from three iPSC lines.

## Data Availability

All data generated or analysed during this study are included in this published article [and its supplementary information files].

## Abbreviations

CDH23: Cadherin 23
CIB2: Calcium and integrin-binding family member 2
CIs: Cochlear implants
HAs: Hearing aids
iPSCs: Induced pluripotent stem cells
LHFPL5: Lipoma HMGIC fusion partner-like 5
MET: Mechanoelectrical transduction
PCDH15: Protocadherin 15
TMIE: Transmembrane inner ear
TMC1: Transmembrane channel-like 1
