# Supplementary material for: Genetic correction of induced pluripotent stem cells from a DFNA36 patient results in morphologic and functional recovery of derived hair cell-like cells
Source: Stem Cell Res Ther. 2024 Jan 2;15:4. doi: 10.1186/s13287-023-03617-9 (PMC10763492; doi:10.1186/s13287-023-03617-9)
Supplement: Supplementary file 1 — Additional file 1. Table S1. Primers. Figure S1. The brightfield images of iPSCs, embryoid and HC-like cells. The morphology of HC-like cells is significantly different from their iPSC lines. Scale bar: 200 μm. Figure S2. Sanger sequencing of HC-like cells. Sanger sequencing of HC-like cells derived from three cell lines showed same gene background with iPSCs. Figure S3. Cell proliferation curve. The A450 on 0, 24, 48 and 72 hours of HC-like cells derived from three iPSC lines. [file 13287_2023_3617_MOESM1_ESM.docx]

**Supplementary material**

Table S1 Primers

| Gene | Forward primer | Reverse primer |
| --- | --- | --- |
| MYO7A | CCTGGAGGCTCTTCTTCCGCAAA | ACCTGTACTCCCCAAACTTGACT |
| POU4F3 | CACCATCTGCAGGTTCGAGT | GGCTTGCTGTTCTTCTCTCGGTA |
| ATOH1 | GCATCCCGTCTACCCGCCTGA | CCAGCTCCGGGGAATGTAGCAAA |
| ESPN | GCCGCTGCCTTCTGTGTCACC | CCACGTCCAGCTGCACTCCC |
| ACTB | TCCTCCTGAGCGCAAGTACTCC | CATACTCCTGCTTGCTGATCCAC |
| TMC1 | AATTCTAAACGTGCATAAAATAGTCATT | GAACAACACTCTTATGGCAAGG |


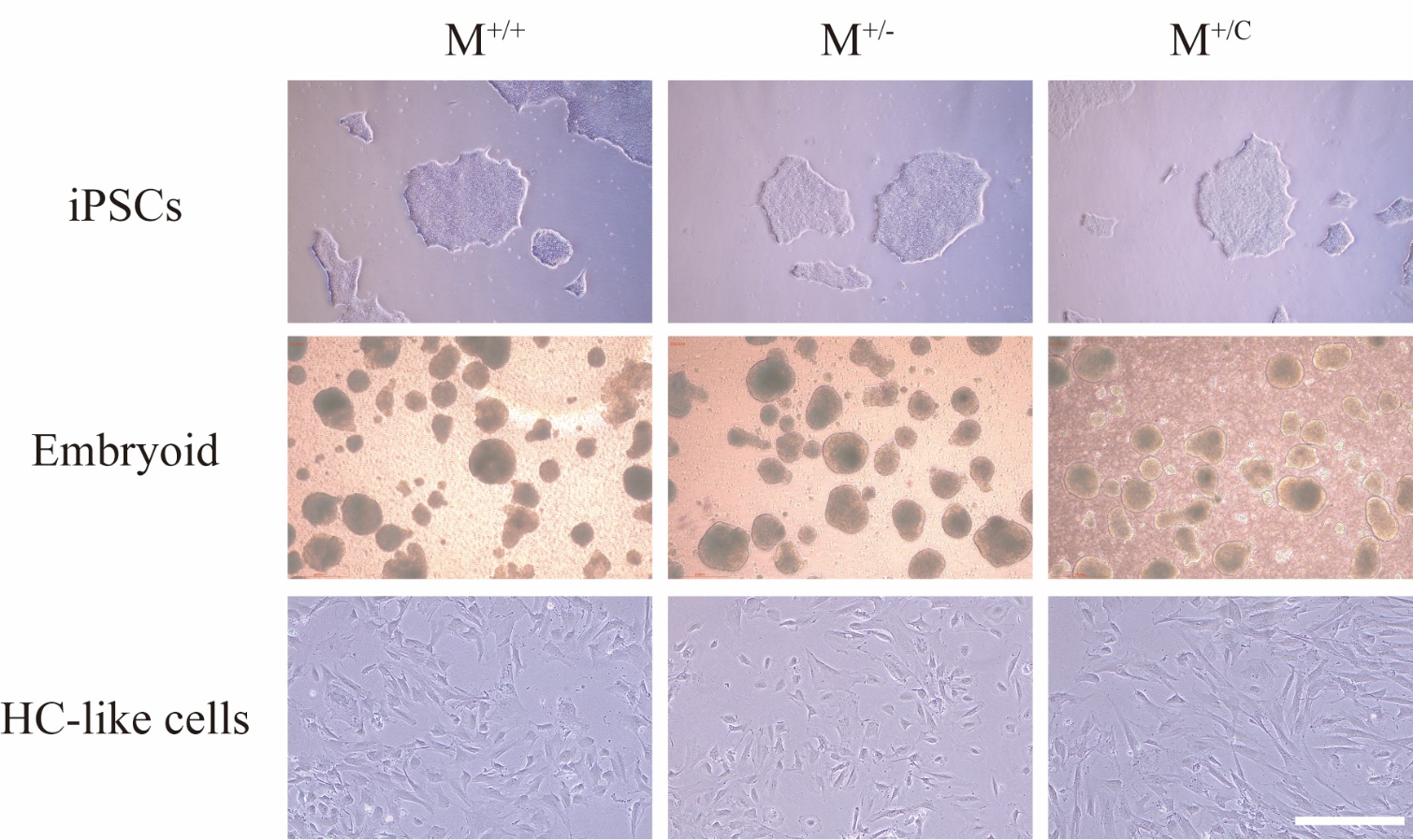


Figure S1. The brightfield images of iPSCs, embryoid and HC-like cells. The morphology of HC-like cells is significantly different from their iPSC lines. Scale bar: 200μm.


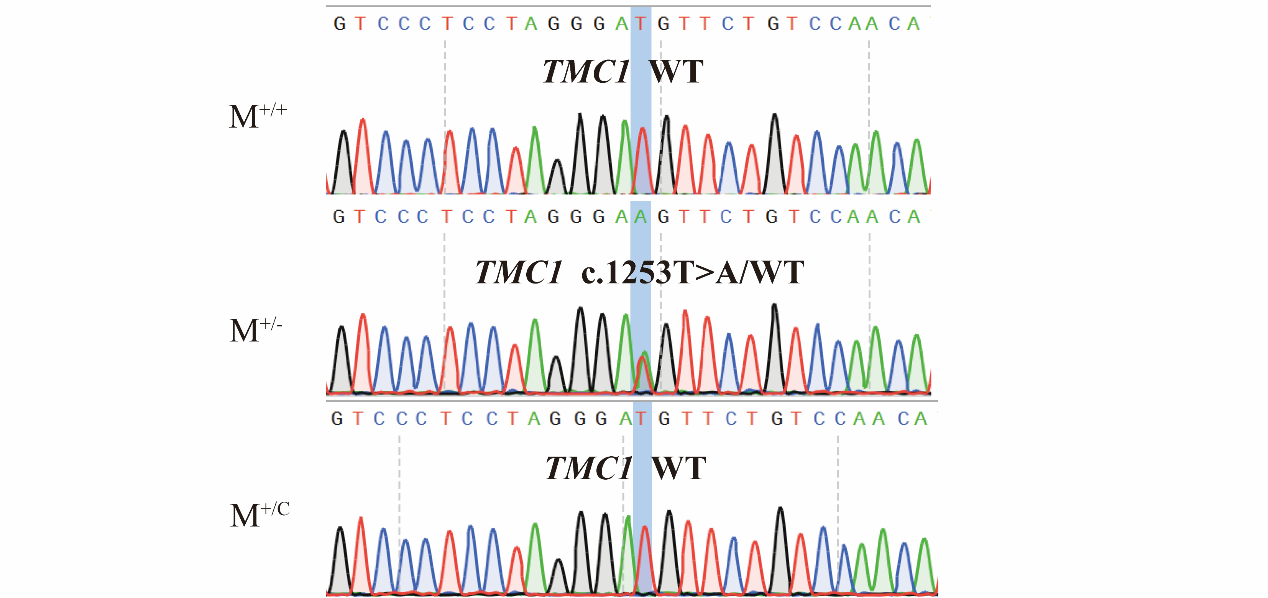


Figure S2. Sanger sequencing of HC-like cells. Sanger sequencing of HC-like cells derived from three cell lines showed same gene background with iPSCs.


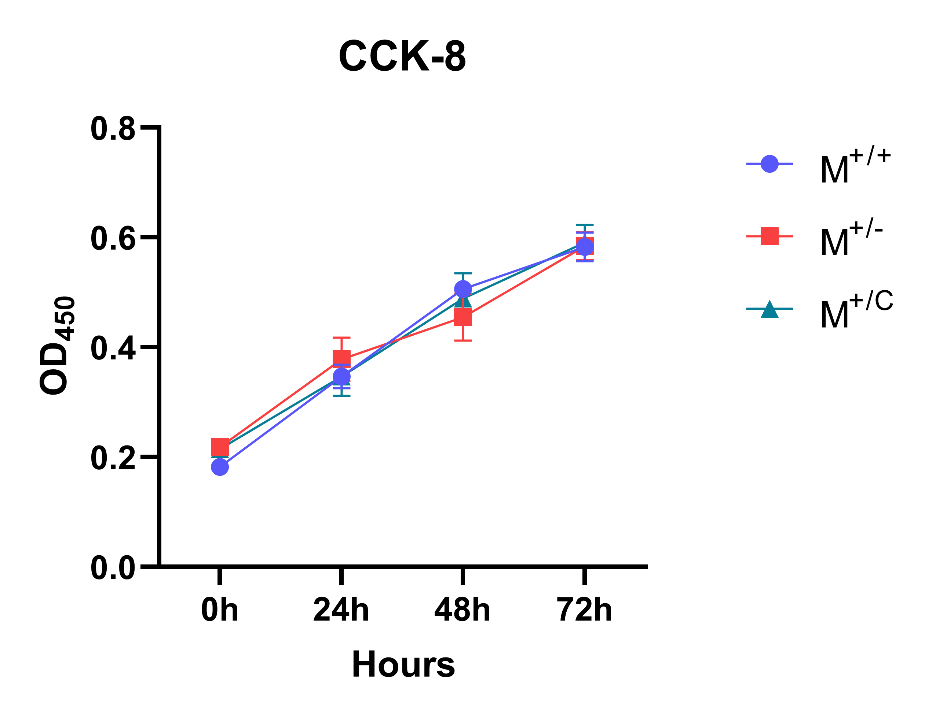


Figure S3. Cell proliferation curve. The A450 on 0, 24, 48 and 72 hours of HC-like cells derived from three iPSC lines.
